# Prostaglandin E1 administration for prevention of contrast-induced acute kidney injury: A systematic review and meta-analysis of randomized controlled trials

**DOI:** 10.1097/MD.0000000000011416

**Published:** 2018-07-20

**Authors:** Ning Geng, Deling Zou, Yanli Chen, Li Ren, Lisheng Xu, Wenyue Pang, Yingxian Sun

**Affiliations:** a Department of Cardiology, Shengjing Hospital of China Medical University; b Department of Cardiology, the First Affiliated Hospital of China Medical University; c Sino-Dutch Biomedical and Information Engineering School, Northeastern University, Shenyang City, Liaoning Province, China.

**Keywords:** angiography, contrast media, nephroprotection

## Abstract

**Background:**

PGE1 has been studied for prevention of CI-AKI in several RCTs and significant heterogeneous results exist.

**Methods:**

We searched PubMed, EMBase, and Cochrane Central Register of Controlled Trials up to December 26, 2017 for RCTs comparing PGE1 with placebo or other active medications for the prevention of CI-AKI in patients. Odds ratio and 95% confidence interval (CI) were used for pooling dichotomous data, while mean difference and 95% confidence interval for pooling continuous data.

**Results:**

Seven RCTs involving 1760 patients were included in this meta-analysis. All these 7 trials reported the incidence of CI-AKI and compared with placebo or other treatment options, PGE1 was associated with a reduced risk of CI-AKI (OR: 0.38, 95% CI: 0.28–0.53; *P* < .001) and only a trend for lower post procedure serum creatinine (Scr) levels compared with control groups at 48 hours (MD: −0.03 mg/dL, 95% CI: −0.08 to 0.02 mg/dL; *P* = .25; 6 trials combined). But the postprocedure Scr levels were significantly reduced in PGE1 groups compared with control groups at 72 hours (MD: −0.07 mg/dL, 95% CI: −0.11 to −0.04 mg/dL; *P* < .001; 4 trials combined). We also meta-analyzed the postprocedure cystatin C (CysC) at 24 and 48 hours with 2 trials. There were lower postprocedure CysC levels in PGE1 groups than those in control groups (MD: −0.18 mg/L, 95% CI: −0.33 to −0.03 mg/L; *P* = .02 at 24 hours and MD: −0.14 mg/L, 95% CI: −0.23 to −0.06 mg/L; *P* = .001 at 48 hours).

**Conclusions:**

PGE1 provides effective nephroprotection against CI-AKI and may act as a part of effective prophylactic pharmacological regimens.

## 1 Introduction

Contrast-induced acute kidney injury (CI-AKI) is a common complication of procedures with intravascular contrast medium administration and is associated with adverse short- and long-term outcomes. As population ages, more patients will be administrated with intravascular contrast media for diagnosis and therapy purposes. Risk factors for CI-AKI, such as chronic kidney disease, diabetes, advanced age, heart failure and anemia,^[1]^ are more common in those patients. Therefore, the incidence of CI-AKI likely will rise.

CI-AKI typically occurs within the first 48 to 72 hours after exposure to intravascular contrast medium administration. Although many of renal impairments are unlikely to be clinically significant, even mild acute kidney injury is associated with increased cost, increased hospital stay, and increased in-hospital and long-term morbidity and mortality.^[2]^ Therefore, we should pay more attention to the prevention of CI-AKI and effective interventions should be developed.

Prostaglandin E1 (PGE1) has been studied for prevention of CI-AKI in several randomized controlled trials (RCTs). There are significant heterogeneous results among those existing trials. The trial conducted by Miao etc. had shown a preventive effect of PGE1 on CI-AKI in older patients (aged ≥70 years) after contrast enhanced computed tomography (CT).^[3]^ A total 370 patients were randomized into PGE1 or control group. The patients in the control group were injected with 100 mL sterile saline and the patients in the PGE1 group with PGE1 (0.4 μg/kg/day) in 100 mL sterile saline before and after iohexol-enhanced (100 mL) CT. The incidence of CI-AKI in the PGE1 group was significantly decreased compared with control group (9.1% vs 22.2%, *P* < .01). While this preventive effect of PGE1 on CI-AKI was not demonstrated in some other trials. Liu et al^[4]^ investigated the preventive effect of PGE1 in 156 patients with mild to moderate renal failure who underwent coronary angiography. The incidence of CI-AKI in the PGE1 plus statins group was only slightly lower than in the statins group, but the reduction was not statistically significant (OR: 0.87, 95% confidence interval: 0.25–2.97). To clarify the disagreement over the effect of PGE1 on the prevention of CI-AKI, we aimed to perform this systematic review and meta-analysis.

## 2 Methods

We conducted this study in accordance with the preferred reporting items for systematic reviews and meta-analysis (PRISMA) checklist.^[5]^ This study is a meta-analysis of RCTs and all data were collected from published trials, so an additional ethical approval is not necessary.

### 2.1 Literature search

We searched PubMed, EMBase, and Cochrane Central Register of Controlled Trials with no language restriction for relevant articles till December 26, 2017 by the PICOS search strategy. Combinations of MeSH terms, entry terms, and text words were used for the search of every theme. For the theme “contrast media,” we used the following key words Contrast Media OR Media, Contrast OR Contrast Materials OR Materials, Contrast OR Contrast Agents OR Agents, Contrast OR Radiocontrast Media OR Media, Radiocontrast OR Radiocontrast Agent OR Agent, Radiocontrast OR Radiopaque Media OR Media, Radiopaque OR Radiocontrast Agents OR Agents, Radiocontrast. For the theme Prostaglandin E1, we used: Prostaglandin E1 OR Lipo-PGE1 OR PGE1 OR Edex OR Viridal OR Prostavasin OR Prostin VR OR Minprog OR Prostine VR OR Vasaprostan OR Caverject OR Sugiran OR Muse OR Alprostadil. Randomized controlled trial OR controlled clinical trial OR randomized OR randomly were used for the search of the theme randomized controlled trial (RCT). For the final search results, we combined the search results of each theme by the Boolean operator “AND.” We also performed manually search for potential eligible studies. Authors of published studies were also contacted for more data as needed. For studies of overlapping patient populations, data from the most informative or most recent publication were included in our meta-analysis.

### 2.2 Inclusion and exclusion criteria

The inclusion criteria were as follows: RCTs including variations on RCT design, such as, quasi- and crossover-RCTs; adult patients undergoing procedures during which the contrast media were needed, irrespective of the basal renal function and comorbidity or comedication; PGE1 with/without other positive drugs were administrated intravenously in experimental group, while placebo with/without other positive drugs were given in control group; there was clear definition of CI-AKI. If CI-AKI was not defined, the patients, whose Scr increased more than 0.5 mg/dL absolutely or 25% relatively, within 3 days were reported; the incidence of CI-AKI and/or levels in serum creatinine (Scr), cystatin C (CysC), or other biomarkers of renal function levels before and after contrast administration were reported in both experimental/control arms; for duplicate data or overlapping studies, only the one most recently published or with the most detailed data was included. The followings were excluded: conference abstracts without needed data; no clear criteria of CI-AKI.

### 2.3 Data extraction

Two reviewers (NG and DZ) independently extracted data from all eligible studies. Disagreements were resolved through discussion with all the reviewers. The extracted information from each trial included: first author; year of publication; sample size; patient characteristics; procedure; interventions in treatment and control groups; type and volume of the contrast medium used; definition of CI-AKI (if there was no prespecified definition, we took a Scr increase more than 0.5 mg/dL absolutely or 25% relatively within 3 days as the definition); incidence of CI-AKI in both PGE1 and control groups; the Scr and/or CysC values before and after contrast medium was applied.

### 2.4 Assessment of risk of bias

Two reviewers (NG and YC) independently assessed risk of bias of each eligible study by creating risk of bias graph and risk of bias summary graph, using the Cochrane Collaboration's tool for assessing risk of bias. This tool evaluated each trial by considering the following sources of bias: selection bias, performance bias, attrition bias, detection bias, reporting bias, and other potential sources of bias. The risk of each bias was evaluated and rated as “low,” “unclear,” or “high” by criteria recommended by the Cochrane Collaboration's tool for assessing risk of bias. Any discrepancy was solved by discussion.

### 2.5 A summary of findings table

A summary of findings table was created for the quality assessment of all outcomes of our meta-analysis across the trials synthesized. Because only RCTs were included, we rated the quality of a body of evidence for every outcome as high primarily and then rated down the quality according to the 5 GRADE considerations (risk of bias, inconsistency, indirectness, imprecision, and publication bias). We used methods and recommendations described in section 8.5 and chapter 12 of the Cochrane Handbook for Systematic Reviews of Interventions^[6]^ using GRADEprofiler software.

### 2.6 Statistical analysis

All the statistical analyses were performed by Review Manager 5.3. Odds ratio (OR) and 95% confidence interval (CI) were used to describe dichotomous data (incidences of CI-AKI), while mean difference (MD) and 95% CI to describe continuous data (the differences of Scr and/or CysC levels between experimental and control groups after contrast media administration) for each study. The heterogeneity across trials was quantified using the *I*^2^ statistic, which indicates the percentage of total variation attributed to statistical heterogeneity rather than chance, with *I*^2^ < 25%, 25% to 50%, and >50% representing mild, moderate, and severe heterogeneity, respectively.^[7]^ We pooled the trials using random-effects model and estimated the absolute between-study variance using the DerSimonian and Laird estimator, considering the potential heterogeneity across included trials due to expected clinical and methodological heterogeneity that might manifest as statistical heterogeneity. Sensitivity analysis was performed to assess the stability of the results by removing a single trial in turn and pooling the remaining ones. *P* < .05 in 2-tailed tests was considered statistically significant.

## 3 Results

### 3.1 Study selection

**Figure 1:**
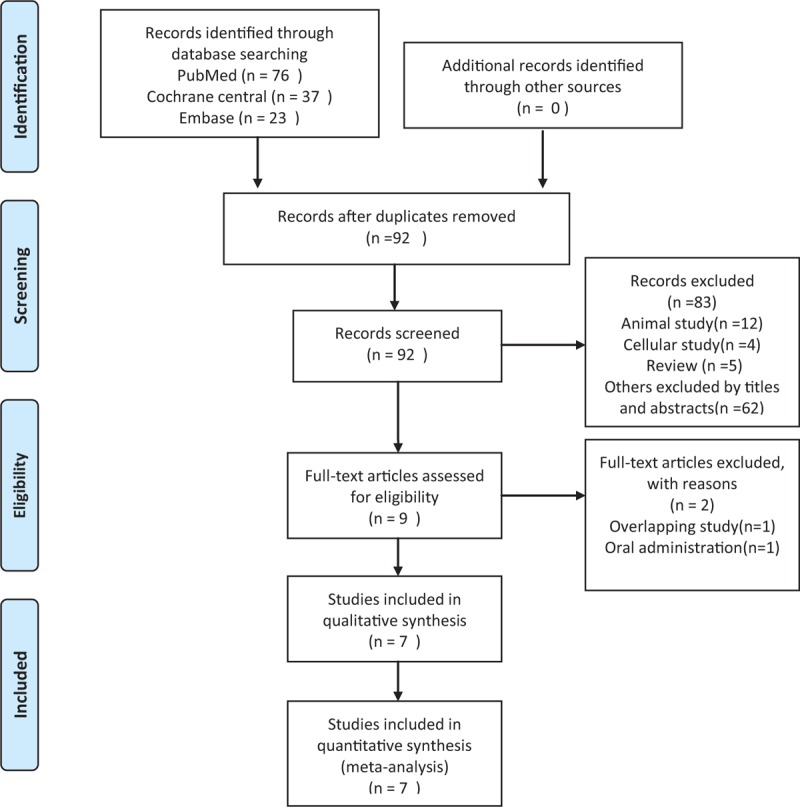
Flow chart for study selection.

We identified 136 potentially relevant citations from the initial search. After removing the duplicates and screening the title and abstract, 9 full-text articles were deemed to be assessed for eligibility. Study by Sketch et al^[8]^ reported overlapping patients with study by Koch et al,^[9]^ but had more informative data. Therefore, Sketch's study was retained for meta-analysis. One study^[10]^ by Franz used oral prostaglandin to assess the renoprotective effect from contrast medium administration and was removed from our analysis according to inclusion criteria. Therefore, 7 randomized controlled studies^[3,4,8,11–14]^ involved 1760 patients undergoing contrast-related procedures were identified and analyzed. Our search strategy and results are outlined in Fig. 1.

### 3.2 Characteristics of included studies

These are presented in Table 1. One study^[8]^ did not report the definition of CI-AKI, but reported the creatinine increase within 48 hours after the administration of contrast medium. Therefore, patients with an increase ≥0.5 mg/dL in Scr level from baseline within 48 hours were considered to suffer from CI-AKI according to inclusion criteria. One study^[3]^ had 2 definitions of CI-AKI based on the changes of Scr and CysC, respectively. We analyzed the patients diagnosed as CI-AKI based on Scr changes. All 7 trials reported the postprocedural Scr values at 48 hours (study Wang et al^[14]^ did not report the mean and standard deviation of postprocedural Scr, so could not be used for the analysis of postprocedural Scr) and 4 trials^[3,4,12,13]^ also reported the postprocedural Scr values at 72 hours.

**Table 1:**
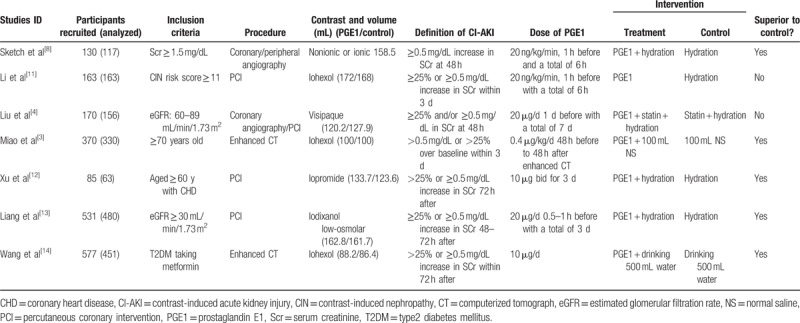
Characteristics of studies included in this meta-analysis.

Two studies had more than 2 arms of studied groups. One^[9]^ had 4 arms (placebo, 10 ng PGE1, 20 ng PGE1, 40 ng PGE1). We took the placebo arm as control group and combined the other 3 arms into one experimental group. For a binary outcome (incidence of CI-AKI), combining the arms simply means adding the numbers of events and total participants over all arms. In case of continuous data, combinations of different arms were carried out by the formulas provided by Rücker et al.^[15]^ The other study^[12]^ had 3 arms (control, hydration, and PGE1 + hydration). We chose the hydration group and the PGE1 + hydration for meta-analysis.

Both nonionic and ionic contrast media were used in 1 study.^[9]^ In the other 6 studies, only nonionic contrast media were used (iohexol, visipaque, iopromide, iodixanol were used in 3, 1, 1, 1 studies, respectively).

Among those 7 RCTs, 2^[3,12]^ enrolled older patients (older than 70 years and 60 years respectively); 2^[4,9]^ enrolled patients with impaired renal function (Scr ≥ 1.5 mg/dL; eGFR: 60–89 mL/min/1.73 m^2^ respectively) and patients with a higher risk developing CI-AKI (CIN risk score ≥ 11) were included in one study.^[11]^ Patients whose eGFR ≤ 30 mL/min/1.73 m^2^ were excluded in 1 study^[13]^ and the remaining study included only patients with diabetes.^[14]^

Contrast medium related procedures were applied in all the studied patients. Enhanced CT was performed in 2 studies,^[3,14]^ coronary/peripheral angiography or percutaneous coronary intervention (PCI) in the other 5 studies.

### 3.3 Patient characteristics

The major characteristics of all the studied population are shown in Table 2. All the baseline characteristics (age, gender, Scr, diabetes, hypertension, BMI) were statistically similar between the PGE1 group and control group in each trial except for that by Sketch et al^[8]^ (not reported).

**Table 2:**
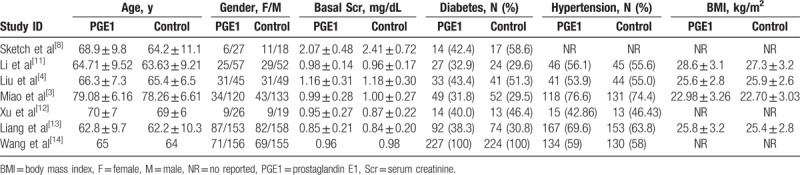
Characteristics of the study patients.

### 3.4 Risks of bias within studies

**Figure 2:**
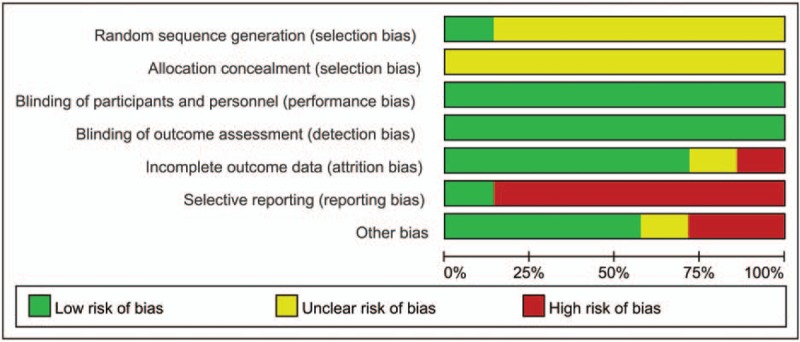
Risk of bias graph.

**Figure 3:**
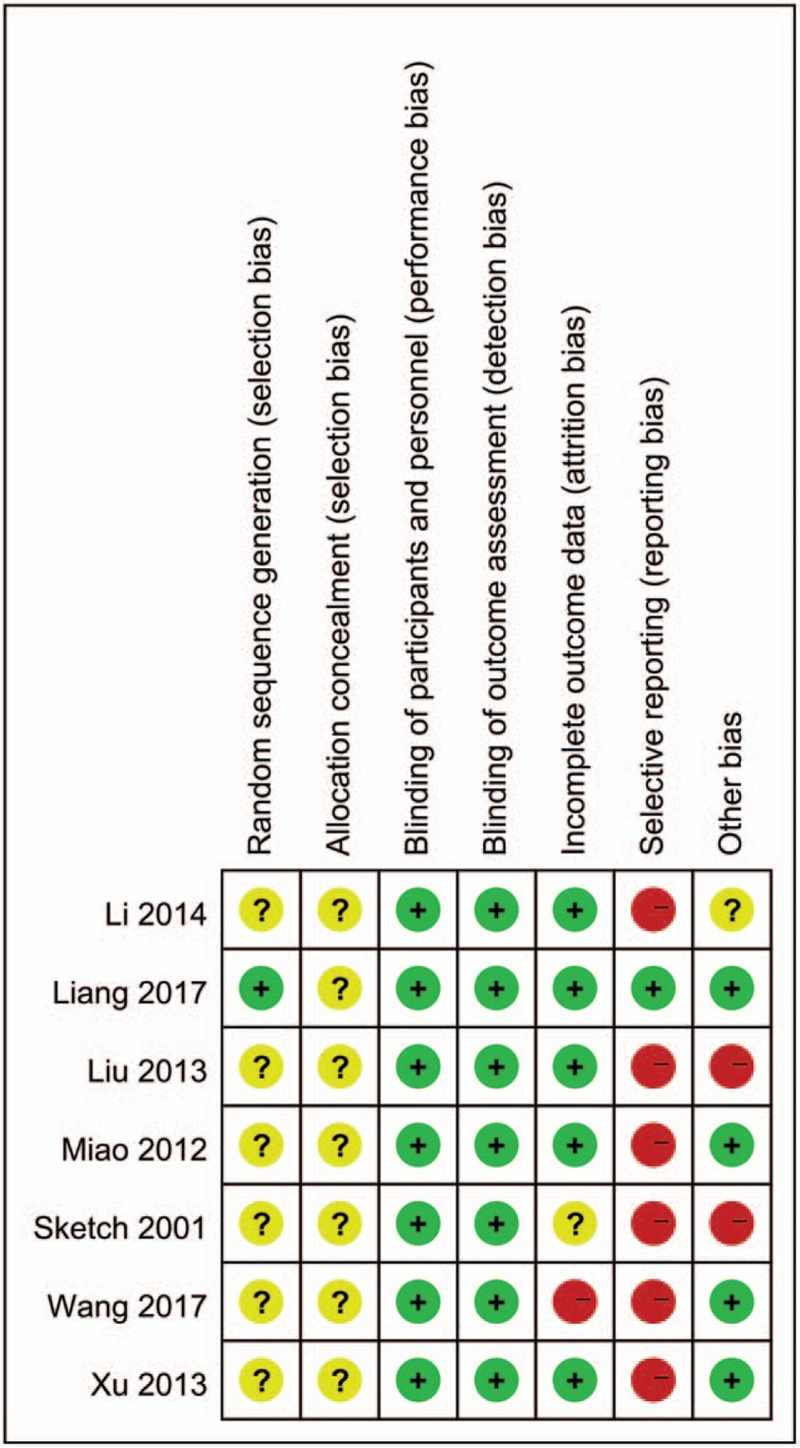
Risk of bias summary graph.

All included studies were randomized, thus minimizing the chances of bias within studies. Risk of bias graph and risk of bias summary graph are presented in Figs. 2 and 3 separately, which evaluated the relevant study characteristics according to Cochrane Handbook for Systematic Reviews of Interventions. Only 1 trial^[13]^ reported method of random sequence generation and none described the concealment of allocation. One study^[8]^ did not describe the definition of CI-AKI in advance.

### 3.5 Incidence of contrast-induced acute kidney injury

Seven RCTs reported data on the incidence of CI-AKI in 1760 patients who had completed the trials and were included in the final analysis. PGE1 was given to 902 patients, whereas 858 patients were in control group and received alternative treatments. The overall incidence of CI-AKI in patients receiving PGE1 was 8.43% compared with 16.43% in the control arms.

**Figure 4:**
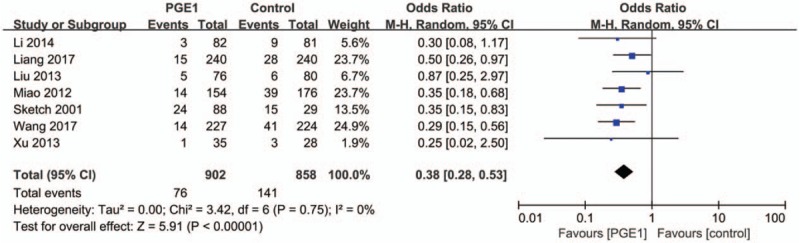
Forest plot showing a decreased incidence of contrast-induced acute kidney injury in the prostaglandin E1 group compared with control group.

In the pooled analysis using a random effects model, patients receiving PGE1 had less risk of CI-AKI compared with the control groups (OR: 0.38, 95% CI: 0.28–0.53; *P* < .001) (Fig. 4). No heterogeneity was present (Tau^2^ = 0.00; Chi^2^ = 3.42, *P* = .75; *I*^2^ = 0%).

Sensitivity analysis of the risk of CI-AKI with PGE1 after 1-by-1 exclusion of each individual study gave effect sizes that were similar in magnitude and direction to the overall estimates (Table 3).

**Table 3:**
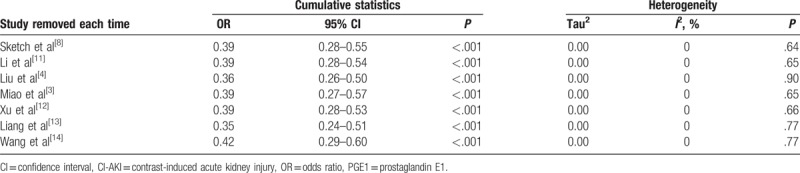
Sensitivity analysis of the risk of CI-AKI with PGE1 after 1-by-1 exclusion of each individual study.

### 3.6 Comparisons of postprocedural Scr levels at 48 and 72 hours between PGE1 and control groups

There were no statistical differences in the baseline Scr and CysC between PGE1 groups and control groups in all included studies except one study by Sketch (not reported). We performed the meta-analyses of the postprocedural Scr differences between PGE1 and control groups at 48 and 72 hours after the contrast medium administration using a random effects model.

**Figure 5:**
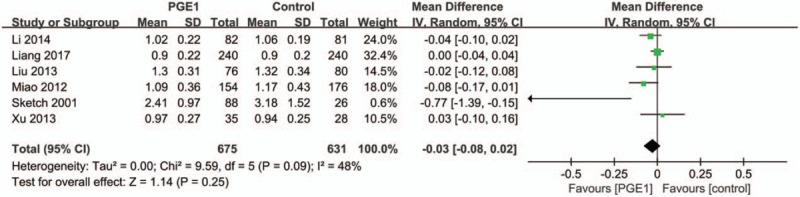
Meta-analysis of the difference of the postprocedure Scr levels between the prostaglandin E1 and control groups at 48 hours.

Based on data provided in 6 trials, the pooled estimate for the MD in 48-hour Scr levels between the PGE1 and control groups was −0.03 mg/dL (95% CI: −0.08 to 0.02 mg/dL; *P* = .25; Fig. 5). This suggested a trend of a less Scr elevation in PGE1 groups compared with control groups, but the difference was not statistically significant.

**Figure 6:**
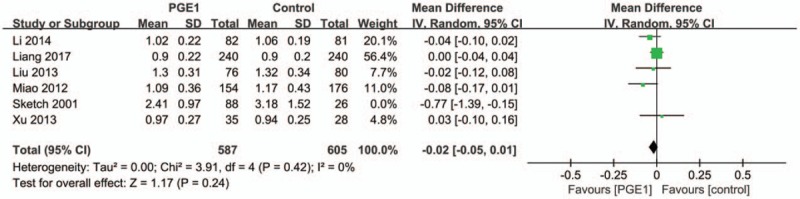
Heterogeneity across studies disappears after removing the study Sketch 2001.

Because of the presence of moderate heterogeneity across studies (Tau^2^ = 0.00; Chi^2^ = 9.59, *P* = .09; *I*^2^ = 48%), we conducted a 1-by-1 exclusion of each individual study to detect the source of heterogeneity. Only after removing the study Sketch 2001 did the heterogeneity across studies disappear (Tau^2^ = 0.00; Chi^2^ = 3.91, *P* = .42; *I*^2^ = 0%) and the MD within 48-hour Scr level was still statistically nonsignificant (pooled MD: −0.02 mg/dL, 95% CI: −0.05 to 0.01 mg/dL) (Fig. 6).

**Figure 7:**
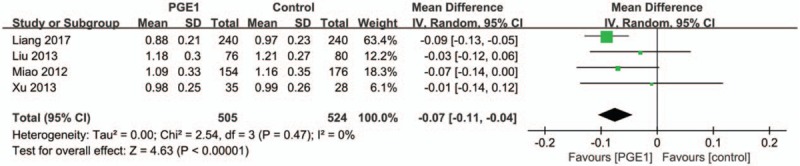
Forest plot demonstrating a decreased postprocedure Scr level in the prostaglandin E1 group compared with control group at 72 hours.

Four trials reported the postprocedural Scr levels at 72 hours after the contrast medium administration. We found that there was a significantly lower Scr level in PGE1 group than in control group. The pooled estimate for the MD in 72-hour Scr levels between the PGE1 and control groups was −0.07 mg/dL (95% CI: −0.11 to −0.04 mg/dL; *P* < .001; Fig. 7) and no heterogeneity was found (Tau^2^ = 0.00; Chi^2^ = 2.54, *P* = .47; *I*^2^ = 0%).

### 3.7 Comparisons of postprocedural CysC levels at 24 and 48 hours between PGE1 and control groups

**Figure 8:**
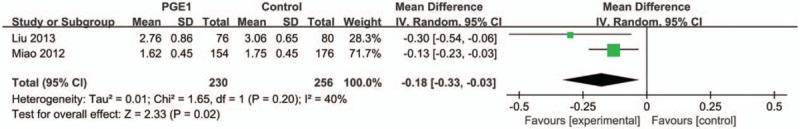
Forest plot demonstrating a decreased postprocedure cystatin C in the prostaglandin E1 group compared with control group at 24 hours.

**Figure 9:**
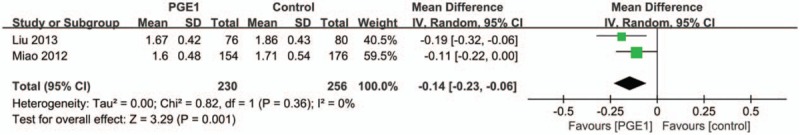
Forest plot demonstrating a decreased postprocedure cystatin C in the prostaglandin E1 group compared with control group at 48 hours.

Among the trials included, only 2 studies^[3,4]^ monitored the serum levels of postprocedural CysC at 24 and 48 hours. We performed the meta analysis of the postprocedural levels of CysC at 24 and 48 hours, and found that there were statistically significant decreases in the levels of postprocedure CysC in PGE1 groups compared with control groups both at 24 and 48 hours (MD: −0.18 mg/L, 95% CI: −0.33 to −0.03 mg/L; *p* = .02 at 24 hours and MD: −0.14 mg/L, 95% CI: −0.23 to −0.06 mg/L; *P* = .001 at 48 hours). No significant heterogeneity was present (Tau^2^ = 0.01; Chi^2^ = 1.65, *P* = .20; *I*^2^ = 40% at 24 hours and Tau^2^ = 0.00; Chi^2^ = 0.82, *P* = .36; *I*^2^ = 0% at 48 hours) (Figs. 8 and 9).

### 3.8 A summary of findings table

We created a summary of findings table for every outcome across the trials involved (Table 4). Outcomes 1 and 2 (preventive effect of PGE1 on CI-AKI and comparation of Scr between PGE1 and control groups at 48 hours) were rated as moderate level that means further research is likely to have an important impact on our confidence in the estimate of effect and may change the estimate. The other outcomes (comparation of Scr between PGE1 and control groups at 72 hours; comparation of CysC between PGE1 and control groups at 24 and 48 hours) were rated as low level that means further research is very likely to have an important impact on our confidence in the estimate of effect and is likely to change the estimate. The reasons for quality lowering were described in the footnote.

**Table 4:**
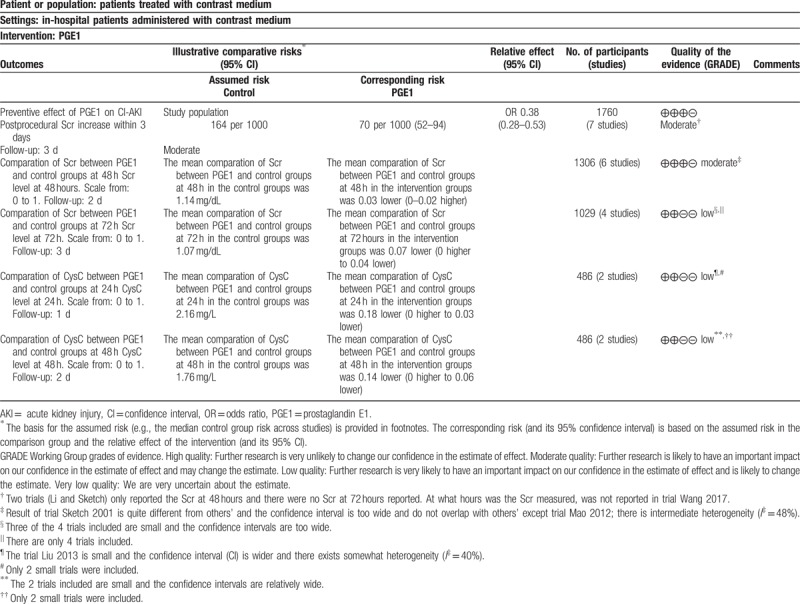
PGE1 for CI-AKI, postprocedural Scr, and CysC level.

## 4 Discussion

CI-AKI represents the third leading cause of hospital-acquired acute kidney injury. The mechanisms of kidney injury caused by contrast media have not been fully elucidated. Both direct toxic injury to the renal tubules and ischemic injury to the renal medulla due to vascular constriction caused by contrast media play an important role in the pathogenesis of CI-AKI.^[16]^

Several strategies have been addressed to mitigate the nephrotoxic effect of contrast agents, but these strategies, except for intravenous hydration, have not yielded encouraging outcomes.^[17]^ Prostaglandins (PGs), hormone-like lipid compounds, have a variety of physiological effects, such as regulating the contraction and relaxation of smooth muscle. Of the PG family, PGE1, also known as alprostadil, is a strong vasodilator and inhibitor of platelet aggregation. So they can be used in the treatment of vascular disorders such as Raynaud's, critical limb ischemia. It is believed that patients with CI-AKI have decreased levels of prostaglandins, causing a shift in physiologic vasoconstriction/vasodilatation balance. Therefore, prophylactic administration of PGE1 might be beneficial in reducing incidences of CI-AKI.^[8]^

In this meta-analysis, we found that the administration of periprocedural PGE1 could cause a reduction in the incidence of CI-AKI (OR: 0.38, 95% CI: 0.28–0.53; *P* < .001).

The criteria for CI-AKI in the included studies were ≥25% and/or ≥0.5 mg/dL increase in Scr within 3 days. But Scr is not an ideal marker of kidney function. Levels of Scr can vary widely depending on a large number of nonrenal factors including age, gender, muscle mass, and hydration status.

In several settings including contrast media exposure, CysC, a protein member of the cysteine proteinase inhibitor family, has been suggested as a sensitive marker of acute kidney injury.^[18]^ Compared with Scr, the serum concentration of CysC is less dependent on age, gender, muscle mass, and nutrition, and therefore more reliably predicts deterioration of renal function.^[19]^ In our pooled analysis, we found that there were statistically significant decrease in the levels of postprocedural CysC, not in Scr, at 48 hours after treatment in PGE1 groups compared with control groups (MD: −0.14 mg/L, 95% CI: −0.23 to −0.06 mg/L; *P* = .001). However, there were only 2 studies included for pooled analysis. Therefore, more RCTs, which employ changes of CysC as the definition of CI-AKI, are called for.

There is a moderate heterogeneity across studies (Tau^2^ = 0.00; Chi^2^ = 9.59, *P* = .09; *I*^2^ = 48%) in our meta-analysis of the postprocedural Scr at 48 hours and only after removing the study Sketch 2001 did the heterogeneity across studies disappear (Chi^2^ = 3.91, *P* = .42; *I*^2^ = 0%), which means study Sketch 2001 was the main source of the heterogeneity. The reasons might be that the baseline Scr values were not reported and both ionic and nonionic contrast media were used in study Sketch 2001.

### 4.1 Limitations

Our meta-analysis of post-procedure Scr values at 48 hours did not show a significant decrease in the PGE1 group compared with the control group (MD: −0.03 mg/dL, 95% CI: −0.08 to 0.02 mg/dL; *P* = .25). This may be caused by a relatively short monitoring period. The Scr monitoring for most included studies was only 48 to 72 hours. CI-AKI can occur beyond 2 days, may not manifest fully up to 5 days.^[20]^ Therefore, some patients developing acute kidney injury beyond 48 to 72 hours might have been missed and the nephroprotective effect of PGE1 may be underestimated. Trials with longer monitoring duration are needed.

Racial differences and genetic polymorphisms may affect efficacy of certain disease processes and medications.^[21]^ Six of all the 7 included trials were performed in China. More studies of the preventive effect of PGE1 on CI-AKI should be conducted in countries other than China.

## 5 Conclusions

In patients undergoing procedures with contrast media administration, periprocedural PGE1 use, compared with placebo or other treatments, reduces the incidence of CI-AKI and is associated with lower postprocedure CysC levels, but not with lower postprocedure Scr levels at 48 hours. More studies, in which postprocedure CysC levels are monitored, are expected.

## Author contributions

**Conceptualization:** Ning Geng.

**Data curation:** Ning Geng, Deling Zou, Yanli Chen, Li Ren, Lisheng Xu, Yingxian Sun.

**Formal analysis:** Ning Geng, Yanli Chen, Lisheng Xu, Wenyue Pang.

**Funding acquisition:** Lisheng Xu.

**Methodology:** Ning Geng, Deling Zou, Wenyue Pang, Yingxian Sun.

**Project administration:** Ning Geng.

**Software:** Ning Geng.

**Supervision:** Ning Geng.

**Writing – original draft:** Ning Geng, Deling Zou, Yanli Chen, Wenyue Pang.

**Writing – review & editing:** Ning Geng, Wenyue Pang, Yingxian Sun.
